# Chronic Kidney Disease-Associated Pruritus in Patients Undergoing Haemodialysis—A Cross-Sectional Study

**DOI:** 10.3390/medicina61060993

**Published:** 2025-05-27

**Authors:** Teng Wang, Jing-Xin Goh, Wubshet Tesfaye, Kamal Sud, Connie Van, Linda Le Do, Surjit Tarafdar, Ronald L. Castelino

**Affiliations:** 1 School of Pharmacy, Faculty of Medicine and Health, The University of Sydney, Sydney, NSW 2006, Australia; twan6108@uni.sydney.edu.au (T.W.); jingxin.goh@sydney.edu.au (J.-X.G.); w.tesfaye@uq.edu.au (W.T.); connie.van@sydney.edu.au (C.V.); linda.do@sydney.edu.au (L.L.D.); 2 School of Pharmacy and Pharmaceutical Sciences, Faculty of Health, Medicine and Behavioural Sciences, University of Queensland, Brisbane, QLD 4072, Australia; 3 Sydney Medical School, Faculty of Medicine and Health, The University of Sydney, Sydney, NSW 2006, Australia; kamal.sud@health.nsw.gov.au; 4 Nepean Kidney Research Centre, Department of Renal Medicine, Nepean Hospital, Kingswood, NSW 2747, Australia; 5 Faculty of Medicine, Western Sydney University, Sydney, NSW 2751, Australia; surjit.tarafdar@health.nsw.gov.au; 6 Department of Renal Medicine, Blacktown Hospital, Western Sydney Local Health District, Blacktown, NSW 2148, Australia; 7 Pharmacy Department, Blacktown Hospital, Western Sydney Local Health District, Blacktown, NSW 2148, Australia

**Keywords:** chronic kidney disease-associated pruritus, prevalence, outcomes, factors, severity

## Abstract

*Background and Objectives*: Chronic kidney disease-associated pruritus (CKD-aP) is a burdensome symptom associated with impaired patient-reported outcomes. There is a paucity of research in this area with unclear aetiology, under-reporting of this symptom, and limited treatment options and management strategies in clinical settings. The objective of this study was to investigate the prevalence of CKD-aP, patient and dialysis-related factors associated with the occurrence of CKD-aP, and the correlation between CKD-aP severity and quality of life, sleep, anxiety, and depression. *Materials and Methods*: This cross-sectional study was conducted in 88 adult (≥18 years) patients undergoing haemodialysis at the outpatient dialysis centre at a major Australian tertiary care university teaching hospital. Demographic- and dialysis-related factors were obtained from electronic medical records and/or patients, while patient outcomes were determined from the self-reported questionnaires; 5-D itch scale, EQ-5D-5L, Patient Health Questionnaire-9, and Beck Anxiety Inventory. We compared demographic, patient-, and dialysis-related factors associated with CKD-aP. *Results*: Out of 88 patients, 67 (76%) agreed to participate in the study. In total, 27 patients (40%) reported having CKD-aP. Most participants experienced moderate CKD-aP severity (*n* = 12), followed by severe or very severe (*n* = 9) and mild (*n* = 6) symptoms. Whilst there was no significant difference in the demographic characteristics, number of medications, dialysis vintage, and Kt/V, a higher number of pruritic participants experienced obstructive sleep apnoea. There was a statistically significant correlation between CKD-aP severity and depression scores (*p* = 0.009). However, there were no significant correlation between CKD-aP and HRQOL (*p* = 0.506). The correlations between CKD-aP severity and outcomes such as sleep and anxiety were also not statistically significant, although they were marginally close (*p* = 0.069 and *p* = 0.095, respectively). *Conclusions*: This study reports a substantial prevalence of CKD-aP reported among patients undergoing HD and the association of severe CKD-aP with depression. Despite the limitation of a small sample size from a single dialysis centre, our findings suggest that the severity of CKD-aP may have implications for patient-reported outcomes. This warrants further investigation in larger-scale studies to better understand the association and optimise outcomes.

## 1. Introduction

Kidney replacement therapy with dialysis (including haemodialysis (HD) and peritoneal dialysis (PD)) or kidney transplantation is offered to patients with advanced (Stage 5) chronic kidney disease (CKD) once they develop intractable symptoms or complications of uraemia. Whilst kidney transplantation leads to resolution of most symptoms and complications, some symptoms and complications improve whilst others may persist or even worsen with commencement of dialysis [1]. Pruritus, commonly referred to as chronic kidney disease-associated pruritus (CKD-aP), is a distressing symptom experienced by patients with advanced CKD and may also occur or worsen after the initiation of dialysis [2,3,4]. The term CKD-aP is also interchangeably referred to as ‘uraemic pruritus’, the pathogenesis of which remains unclear and several hypotheses have been proposed including buildup of one or more uraemic toxins, derangements of the immune system, or hyperactivation of the opiodergic system [2]. One study proposed a multifactorial mechanism that highlights complex interactions between dermal mast cells, nerve fibres, epidermal keratinocytes, and TH1 lymphocytes [3]. CKD-associated pruritus has also been linked to elevated concentrations of β2-microglobulin (a pruritogenic factor); upregulation of kappa-opioid receptors, which have an antipruritic effect; and various cytokines secreted by TH1 lymphocytes, which may significantly contribute to the pathogenic pathways [3]. It is also increasingly recognised that a range of additional factors may contribute to or be associated with CKD-aP. These can be broadly categorised into dialysis-related and biochemical factors. Dialysis-related factors include dialysis dose, dialysis vintage, hemodialyzer membrane, and dialysis modality, while biochemical factors include hyperparathyroidism, hyperphosphatemia, and hypercalcemia [2,5,6]. Consequently, current interventions for CKD-aP generally aim to address these contributory factors to alleviate symptoms and may include the use of vitamin D derivatives, calcimedins, topical emollients, gabapentinoids, antihistamines, opioid agonists, and phototherapy. These treatment strategies have resulted in limited success in providing long-term relief and applicability of these treatments is also hampered by adverse effects and a dearth of high-quality evidence supporting their efficacy [7,8,9,10,11]. However, emerging evidence supports the use of Difelikefalin, a selective agonist of kappa opioid receptors with antipruritic effects. It has shown to significantly reduce pruritus intensity and improve pruritus-related quality of life [12].

Nevertheless, CKD-aP continues to remain a pertinent healthcare concern for both patients and providers, due to its adverse implications on patient-reported outcomes including reduced quality of life, impaired sleep, depression, and anxiety [13]. Furthermore, CKD-aP is also associated with increased medical burden, as evidenced by heightened utilisation of medications, increased hospitalisations, and higher mortality [4,14].

The limited awareness surrounding CKD-aP can be attributed in part to underreporting by patients, which, compounded by the limited long-term success to treat this condition, amplifies the adverse impact on patient-reported outcomes [15]. Therefore, it is imperative to conduct additional studies to gain a better understanding of CKD-aP. The objective of this study was to investigate the prevalence of CKD-aP in patients on HD, and identify demographic-, clinical-, and dialysis-related factors that are associated with the occurrence of CKD-aP. Furthermore, this study aimed to evaluate the associations between CKD-aP severity and patient-reported outcomes, including sleep quality, health-related quality of life, anxiety, and depression.

## 2. Materials and Methods

### 2.1. Study Design, Participants, Sampling, and Setting

This cross-sectional study was conducted in an outpatient dialysis facility of a tertiary care university teaching hospital in metropolitan New South Wales, Australia. The outpatient dialysis facility includes 22 chairs and provides maintenance HD services to 88 adult (≥18 years) patients. All patients, after their first few haemodialysis sessions, receive haemodiafiltration using FX^®^ CorDiax dialyzers as the default dialyzer. These dialyzers use Helixone^®^ plus membrane designed to improve middle molecule clearance that may play a role in CKD-aP. A purposive sampling was employed targeting all patients receiving dialysis at this centre, with data collection conducted throughout the month of October 2023, spanning a 4-week period. Patients who had CKD but were not undergoing dialysis, as well as those receiving kidney supportive care/end of life care, were excluded from the study. This study was approved by the Institutional Human Research Ethics Committee (2023/PID01393).

### 2.2. Study Instruments

This study utilised a set of validated instruments that had been previously employed in patients with kidney disease to ensure compatibility with existing literature. We used electronic medical records to obtain baseline information from participants who provided consent to participate in the study. Participants also received a self-reported questionnaire to report any missing demographic information (to capture missing data from the electronic medical records), including their age, gender, education, marital status, and smoking status. If a participant reported CKD-aP during the initial screening, subsequent self-reported questionnaires were provided to ascertain assessed outcomes of CKD-aP severity, quality of life, sleep quality, depression, and anxiety.

The evaluation of CKD-aP was carried out using the 5-D Itch scale, which self-assessed the duration, degree, direction, disability, and distribution aspects of itching [16]. The Cronbach’s alpha for the 5-D Itch Scale has been previously reported to range from 0.73 to 0.99, indicating satisfactory internal consistency [17,18,19]. CKD-aP severity was categorised based on the scores: ≤8 for no pruritus, 9–11 for mild pruritus, 12–17 for moderate pruritus, 18–21 for severe pruritus, and ≥22 for very severe pruritus [20].

Health-related quality of life (HRQOL) was assessed using the EQ-5D-5L, a standardised questionnaire comprising five dimensions: mobility, self-care, usual activities, pain/discomfort, and anxiety/depression [21]. The Cronbach’s alpha values of EQ-5D-5L were consistently reported between 0.79 and 0.85 in previous studies [22,23,24]. Index scores range from −0.3 to 1, where 1 is the most optimal state of health. Furthermore, anxiety and depression were also individually assessed using the Beck Anxiety Inventory and Patient Health Questionnaire-9 (PHQ-9), respectively [25,26]. These instruments assessed the severity and frequency of common symptoms associated with anxiety and depression experienced by participants. In previous studies, the Cronbach’s alpha for the Beck Anxiety Inventory and PHQ-9 were reported to be 0.9 and 0.8, respectively, indicating good internal consistency [27,28]. Anxiety was classified according to the total score where a range of 0–7 is considered minimal, 8–15 is mild, 16–25 is moderate, and 26–63 is severe extent [29]. The PHQ-9 scoring system classifies depression severity from none to minimal (0–4), mild (5–9), moderate (10–14), moderately severe (15–19), and severe (20–27). The Insomnia Severity Index (ISI), a 7-item questionnaire, was utilised to capture the symptoms of insomnia experienced by patients [30]. This tool is a 5-point Likert scale ranging from 0 for no problem to 4 for very severe problem, providing a total summative score spanning from 0 to 28. This is, in turn, categorised into: lack of insomnia (0–7), sub-threshold insomnia (8–14), moderate insomnia (15–21), and severe insomnia (22–28) [30].

### 2.3. Data Collection

The recruitment of study participants involved approaching patients during their HD sessions and inquiring about their interest in participating in the study. Those who expressed willingness to participate were then provided with a participant information sheet and consent form. Upon obtaining consent, participants were guided to complete the multiple questionnaires ascertaining information pertinent to sociodemographic characteristics, presence of CKD-aP and its severity, sleep quality, quality of life, depression, and anxiety. The quality of life of those without CKD-aP was also assessed. Data pertaining to participant’s medical conditions, medication history, the use of phosphate binders and serum phosphate levels, dialysis vintage, and Kt/V were obtained through electronic medical records and pathology results. Medication history was ascertained by confirming with patients and contacting their local pharmacies, as required. This included both prescription medications and over-the-counter products. Single and independent data entry was carried out using Microsoft Excel 365 and stored in a password-protected file on the shared drive of the pharmacy department at the hospital. Access to the data was restricted to contributing authors. After the completion of the analysis, the data remained de-identified to ensure confidentiality.

### 2.4. Outcomes

The primary outcome of this study was the prevalence of CKD-aP among patients undergoing HD. Secondary outcomes encompassed the presence and scales of insomnia, anxiety, depression, and HRQOL to investigate its association with the severity of CKD-aP. In addition, this study sought to explore factors that were associated with the presence of CKD-aP.

### 2.5. Statistical Analyses

Single and independent data entry was carried out using Microsoft Excel 365, The data on excel were imported to IBM SPSS statistics 28.0.0.0 for statistical analysis. The primary objective was to ascertain the prevalence of CKD-aP, which was presented as a proportion of patients experiencing various degrees of pruritus. A chi-square test was used to identify categorical variables associated with CKD-aP, while continuous variables like age were analysed using Spearman’s correlation analysis. The relationship between CKD-aP severity with HRQOL, sleep quality, depression, and anxiety severity were also analysed using Spearman’s correlation analysis. The comparison of quality of life, dialysis vintage, and Kt/V between patients with or without CKD-aP was carried out using the non-parametric Mann–Whitney U test. The association between CKD-aP severity and a range of patient-reported outcomes was performed using the non-parametric Kruskal–Wallis Test. In our analysis, we grouped patients reporting ‘severe’ and ‘very severe’ symptoms in one category for better data distribution. Statistical significance was set at a two-sided *p*-value of <0.05 for all analyses.

## 3. Results

### 3.1. Study Sample

Out of 88 patients, 67 (76%) agreed to partake in this study. The baseline characteristics of the patients are presented in Table 1. Gender distribution showed a slight preponderance of males (67%) compared to females (33%). Common comorbidities in the participants included hypertension (73%), coronary artery disease (63%), and diabetes (60%). Obstructive sleep apnoea (OSA) was present in a notable percentage of participants (9%), and other conditions such as depression/anxiety (15%) were also observed. Medical comorbidities, medications, and dialysis-related characteristics are presented in Table 2.

### 3.2. Prevalence of Pruritus and Associated Factors

A total of 27 participants reported experiencing CKD-aP, constituting a prevalence of 40%. The median age of those with pruritus was 62 years (IQR 53.5–72), compared to 66 years (IQR 53.5–72) for those without pruritus, although this was statistically not different. A significant majority of those affected by CKD-aP were male (67%) and had an average dialysis vintage of 37 months (vs. 53 for those without pruritus), although this was not statistically significant. The Kt/V values of patients in both groups were also similar.

### 3.3. Correlations Between CKD-aP Severity and Patient Outcomes

**Figure 1:**
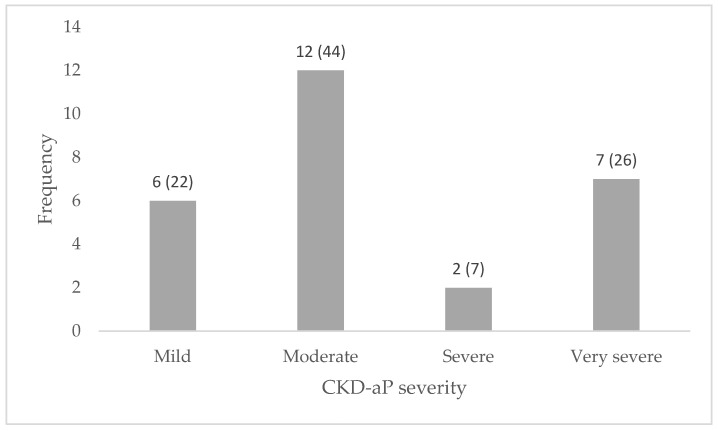
Distribution of self-reported CKD-aP severity among study population. Frequency of severity are given as number (percentage).

The assessment of CKD-aP severity within the study population revealed that most participants experienced moderate CKD-aP severity (*n* = 12) followed by very severe (*n* = 7), mild (*n* = 6), and severe symptoms (*n* = 2). CKD-aP severity distribution is summarised in Figure 1. Our analysis revealed a statistically significant correlation between CKD-aP severity and depression scores (*p* = 0.009). However, there was no correlation between CKD-aP and HRQOL (*p* = 0.506). The correlations between CKD-aP severity and outcomes such as sleep and anxiety were also not statistically significant, albeit marginally (*p* = 0.069 and *p* = 0.095, respectively). The correlation of CKD-aP severity with patient outcomes is presented in Table 3.

## 4. Discussion

This study presents findings on the prevalence of CKD-aP in HD patients at an Australian satellite HD centre, factors associated with CKD-aP occurrence, and correlation between CKD-aP severity and patient-reported outcomes. We found a CKD-aP prevalence of 40% within our HD study population, and more importantly, a significant association of severity of CKD-aP with depression and a non-significant association with poorer sleep quality and anxiety.

The 40% prevalence of CKD-aP in our HD population aligns closely with prior studies that investigated the occurrence of CKD-aP in HD cohorts. A meta-analysis of 42 cross-sectional studies reported a pooled CKD-aP prevalence of 55% among HD patients since 2002 [31]. Discrepancies in prevalence may be attributed to several factors, including the adoption of advanced dialysis technology, such as hemodiafiltration using high-flux biocompatible dialysis membranes utilised in our study setting. Notably, the employment of high-flux membranes has demonstrated a reduction in the incidence of CKD-aP when compared to low-flux dialysers [32,33,34]. Previous research has identified a significant association between the use of low-flux membranes and the exacerbation of CKD-aP, underscoring the potential of high-flux membranes to mitigate the incidence and severity of CKD-aP [33]. This can be ascribed to the enhanced efficiency of high-flux membranes in removing both low and middle molecular weight molecules, in contrast to low-flux membranes, which primarily clears low molecular weight solutes [35].

Whilst various HD-related factors contribute to CKD-aP prevalence, some studies have also reported significant variations in the prevalence of CKD-aP between dialysis modalities. One study exhibited a higher prevalence of CKD-aP in PD patients compared to those on HD (62.6% vs. 48.3%), with a 1.8-fold increased risk [36]. The aetiology is unclear, but it may be associated with the accumulation of middle molecular weight uremic toxins including β 2-microglobulin, which has a higher clearance in high-flux HD compared to PD [36,37]. In contrast, a Taiwanese study similarly reported a lower CKD-aP prevalence in PD compared to HD (38%) [38]. This variation may be attributed to greater residual kidney function observed in PD patients, which corresponds to improved clearance of uremic toxins, enhanced preservation of erythropoietin production, and maintenance of calcium, phosphorus, and vitamin D homeostasis [38,39]. In our study, CKD-aP was not associated with small solute clearance with dialysis (Kt/V), supporting the hypothesis that accumulation of middle molecules may play a role in CKD-aP aetiology. Further, regarding the association between CKD-aP and inflammation, patients on PD exhibit reduced inflammation compared to those on HD, with lower levels of inflammatory mediators such as Interleukin-6 and C-reactive protein (CRP) [38,40]. These discrepancies highlight the complexity of factors influencing the prevalence of CKD-aP across patient factors and dialysis modalities which require further elucidation.

Previous studies have found higher C-reactive protein (CRP) levels are associated with heightened CKD-aP severity [41]. Additionally, rather than OSA being a correlate for pruritus, it has been reported that patients with pruritus had higher CRP levels, which serves as a biomarker for disease activity in OSA [42,43]. Other medical risk factors and correlates of CKD-aP reported in the literature, but not found to be significant in this study, include high serum phosphate, hypertension, and diabetes [15,42,43,44]. Although our findings showed no correlation between gender and CKD-aP, large-scale studies have demonstrated that males are at a higher risk of experiencing CKD-aP [4,45]. Whilst some studies have also suggested that being female may predispose one to CKD-aP [33,46], or that there is no overall association between gender and prevalence [47], these findings were limited by either the studies’ small sample size, unadjusted analysis or imbalanced gender ratio. The correlation between gender and CKD-aP prevalence may be explained by established relationships between gender and itch-related mechanisms, as well as the impact of gender on the opioid–cannabinoid system and neurokinin 1 systems [45]. Our study found the median age of patients reporting CKD-aP symptoms are younger than those without CKD-aP which aligns with findings that younger patients are more likely to report CKD-aP, with age < 70 significantly associated with CKD-aP [15,42,46,48].

Our findings indicate CKD-aP’s association with depression was contingent on CKD-aP severity. Whilst this study found no significant association between CKD-aP severity and quality of life, anxiety, or sleep quality, multiple studies have reported associations between CKD-aP severity and depressive symptoms, as well as quality of life and sleep quality [46,49,50,51,52,53]. Therefore, the absence of significant associations in our findings does not eliminate the potential association of CKD-aP severity on anxiety and sleep quality. It has also been identified that the severity of CKD-aP is an independent predictor of mortality, however, this was not within the scope of our study [39,44,54,55]. The observed associations underscore the importance of proactive screening for and routine assessment of CKD-aP in patients on HD, while ensuring that all modifiable factors such as dialysis dose, solute clearances, dialyzer membrane type, and biochemical abnormalities such as hyperparathyroidism, hyperphosphatemia, and hypercalcemia have been adequately addressed. Recognising these links may support earlier intervention and the use of emerging therapies like difelikefalin to reduce symptom burden and improve patient-reported outcomes, including depression.

This study possesses several notable strengths, including access to one of the largest dialysis centres in Australia, and the use of multiple questionnaires to capture multidimension healthcare outcomes. The selected questionnaires employed also minimised recall bias by focusing on shorter recall periods (e.g., the past two weeks). However, our study also has some limitations. Firstly, the findings of this study may have limited generalisability since it was conducted at a single dialysis centre with a HD cohort only targeting a small sized patient population. The small sample size, especially when exploring the link between CKD-aP and patient-reported outcomes, may have affected the reported findings, highlighting an important research gap for future studies. Additionally, the use of purpose sampling may have introduced selection bias, which could limit the representativeness of the sample and further affect the generalisability of the results, thus, potentially limiting our findings’ applicability to other dialysis centres with differing patient characteristics or alternative modalities like PD. Furthermore, due to the nature of the cross-sectional study and data collection at a single time point, there is no longitudinal follow-up data to examine associations between changes in CKD-aP severity and patient outcomes (including mortality) over time.

## 5. Conclusions

This cross-sectional study found CKD-aP prevalence in an HD cohort in Australia to be 40%. The severity of CKD-aP was also found to have a significant association with patient outcomes like depression. However, further studies should be conducted on a larger scale, encompassing multiple dialysis centres and various dialysis modalities to explore predictive factors for both the incidence and severity of CKD-aP and its association with clinical outcomes.

## Figures and Tables

**Table 1:** Demographic characteristics of the study participants.

Variables	Overall (*n* = 67)	Presence of Pruritus		*p* Value
		Yes (*n* = 27)	No (*n* = 40)	
Median age, years (IQR)	65 (53.5–72)	62 (53.5–72)	66 (53.5–72)	0.61
Gender, *n* (%)				1.00
Male	45 (67)	18 (67)	27 (68)	
Female	22 (33)	9 (33)	13 (33)	
Education, *n* (%)				0.47
Primary	7 (10)	3 (11)	4 (10)	
HS diploma	25 (37)	9 (33)	16 (40)	
VET	18 (27)	7 (26)	11 (28)	
Bachelor’s	15 (22)	6 (22)	9 (23)	
Master’s	2 (3)	2 (7)	0 (0)	
Smoking status, *n* (%)				0.46
Non-smoker	31 (46)	13 (48)	18 (45)	
Smoker	5 (7)	3 (11)	2 (5)	
Ex-smoker	31 (46)	11 (41)	20 (50)	

Abbreviations: HS, high school; VET, vocational education training. Results are given as number (percentage) or median (interquartile range).

**Table 2:** Medical comorbidities, medications, and dialysis-related characteristics of the study participants.

Variables	Overall (*n* = 67)	Presence of Pruritus		*p* Value
		Yes (*n* = 27)	No (*n* = 40)	
Medical factors, *n* (%)				
Diabetes	40 (60)	15 (56)	25 (63)	0.61
Coronary artery disease	42 (63)	16 (59)	26 (65)	0.68
Hypertension	49 (73)	17 (63)	32 (80)	0.13
Heart failure	9 (13)	3 (11)	6 (15)	0.67
Obstructive sleep apnoea	6 (9)	5 (19)	1 (5)	0.01 *
Depression/anxiety	10 (15)	4 (15)	6 (15)	1.00
Obstructive lung disease	9 (13)	3 (11)	6 (15)	0.67
Osteoporosis	4 (6)	3 (11)	1 (3)	0.15
Osteoarthritis	5 (7)	1 (4)	4 (10)	0.35
Hypercholesterolemia	42 (63)	16 (59)	26 (65)	0.68
Gastroesophageal reflux	31 (46)	14 (52)	17 (43)	0.43
Gout	13 (19)	7 (26)	6 (15)	0.26
Atrial fibrillation	5 (7)	3 (11)	2 (5)	0.35
Hypothyroidism	7 (10)	4 (15)	3 (8)	0.33
Anaemia	52 (78)	23 (85)	29 (73)	0.17
Phosphate binder use	48 (72)	19 (70)	29 (73)	0.90
Phosphate serum levels (mmol/L)	1.67 (1.38–1.90)	1.59 (1.34–2.07)	1.67 (1.40–1.88)	0.67
Parathyroid hormone levels (pmol/L)	47.8 (26.9–79.8)	53.4 (27.4–125.0)	46.5 (23.7–76.0)	0.49
Number of medications	11.5 (8.0–14.0)	12.0 (8.0–13.0)	11.0 (8.5–14.0)	0.71
Dialysis vintage (months)	52.0 (29.0–80.0)	37.0 (23.0–65.0)	53.5 (36.5–83.7)	0.17
Kt/V	1.78 (0.35)	1.71 (0.37)	1.83 (0.33)	0.15

Results are given as number (percentage) or median (interquartile range). * significant association.

**Table 3:** Correlations between CKD-aP severity and different patient outcomes.

Outcomes	Overall Median (IQR) *	*p* Value
Sleep quality	14.0 (8.0–22.0)	0.069
Anxiety	13.00 (9.25–26.00)	0.095
Depression	9.50 (5.75–16.00)	0.009
Quality of life (EQ-5D-5L)	0.81 (0.47–0.93)	0.506

* The self-reported index scores were calculated based on the median of the total scores completed by the 27 participants with CKD-aP.

## Data Availability

The datasets generated and analysed are available upon request.

## Abbreviations

The following abbreviations are used in this manuscript:

CKD	Chronic kidney disease
CKD-aP	Chronic kidney disease-associated pruritus
CRP	C-reactive protein
eGFR	Estimated glomerular filtration rate
HD	Haemodialysis
HRQOL	Health-related quality of life
HS	High school
OSA	Obstructive sleep apnoea
PD	Peritoneal dialysis
PHQ-9	Patient health questionnaire-9
VET	Vocational education training
WSLHD	Western Sydney Local Health District
